# Acute Cholecystitis in a Patient With Situs Inversus

**DOI:** 10.7759/cureus.60172

**Published:** 2024-05-12

**Authors:** Sumayah A Althunayan, Nawaf S AlRubaysh, Jehad A Alshaban, Salah O Ali

**Affiliations:** 1 General Surgery, Buraidah Central Hospital, Buraidah, SAU; 2 General Practitioner, King Fahad Specialist Hospital, Buraidah, SAU

**Keywords:** gallstones, hypochondriac tenderness, laparoscopic cholecystectomy, autosomal recessive genetic disorder, acute cholecystitis, situs inversus totalis

## Abstract

A rare disorder called situs inversus partialis (SIP) is characterized by the transposition of organs in the abdomen or thoracic cavity from one side of the body to the other (the mirror image of normal). Autosomal dominant, autosomal recessive, rare genetic mutations, and X-linked recessive inheritance patterns have been identified to be involved in this condition. Laparoscopic cholecystectomies have been successfully performed on patients with SIT. Due to challenges in spatial orientation and the identification of anatomical variations brought on by the abdominal organs' mirror image, surgery is more complicated and takes longer. We describe a 40-year-old female case who had acute cholecystitis. Laparoscopic cholecystectomy was used to treat this patient, a highly effective procedure for both the treatment and care of these patients. Post-surgical examination and follow-up revealed improvement in the patient's condition without subsequent complications.

## Introduction

The unusual congenital disorder known as situs inversus is defined by the translocation of organs to the body's opposing side [1]. Although patients with this condition can present with cholelithiasis and cholecystitis at rates comparable to those seen in the general population, their signs and symptoms can be deceiving because of their altered anatomy [2-4]. Acute cholecystitis refers to the inflammation of the gallbladder, predominantly caused by the obstruction of the cystic duct by gallstones. Clinically, it manifests as severe right upper quadrant abdominal pain, often radiating to the back or right shoulder, accompanied by nausea, vomiting, fever, and leukocytosis. Typically, Murphy's sign, characterized by tenderness in the right upper quadrant upon inspiration during palpation, is elicited. Situs inversus is a rare congenital condition characterized by the mirror-image transposition of thoracic and/or abdominal organs. It is classified into the following two types: situs inversus totalis (SIT), where all organs are mirrored, and situs inversus partialis (SIP), where only specific organs are transposed [4]. Clinical signs of situs inversus include dextrocardia, where the heart is located on the right side, and liver dullness on the left side. Patients may also present with unusual patterns of pain or discomfort due to the reversed anatomy, leading to potential diagnostic challenges, as illustrated by acute cholecystitis masquerading as left-sided abdominal pain in SIP patients. SIT mostly happens in 90% of all SI cases, so SIP is a very rare condition [5].

When a patient has SI, the common causes of acute abdominal discomfort in the general population can result in a misdiagnosis. For instance, appendicitis may cause discomfort in the left iliac fossa, causing it to be mistakenly diagnosed as acute diverticulitis. Cholecystitis can cause pain in the left upper quadrant, which makes it simple to mistake for gastritis [6]. Although acute cholecystitis is among the most prevalent illnesses needing surgical care, it might be challenging to diagnose correctly in patients with situs inversus. Due to their state, when surgery is required, a more complex technique and advanced surgical abilities are necessary to tackle these uncommon conditions [7]. The most common treatment for cholecystitis is laparoscopic cholecystectomy, which is easily accomplished. Laparoscopic cholecystectomy has allegedly been used to treat some instances of SIT [8]. However, this procedure is still complicated, particularly for right-handed surgeons [9].

The aim of this study is to investigate and report on a rare occurrence of acute cholecystitis in a patient with situs inversus partialis (SIP). The primary objective is to elucidate the diagnostic challenges associated with acute cholecystitis in individuals with SIP due to their altered anatomy, which can lead to atypical clinical presentations. Furthermore, the study seeks to underscore the importance of recognizing situs inversus and its variants in clinical practice, particularly in the context of acute abdominal conditions such as cholecystitis. By presenting a detailed case report of a 40-year-old female patient who underwent laparoscopic cholecystectomy for acute cholecystitis complicating SIP, this study aimed to contribute to the existing literature on this rare clinical scenario and provide insights into the optimal diagnostic and management strategies for similar cases in the future.

## Case presentation

A 40-year-old female known to have hypothyroidism and situs inversus state arrived at the hospital complaining of abdominal pain, vomiting, and sweating. The pain was located in the epigastric and left upper quadrant (LUQ) areas, radiating to the back, related to fatty food intake, and associated with multiple episodes of vomiting. The patient claimed no history of fever, no change in bowel habits, and no other associated obstructive symptoms were found.

**Figure 1:**
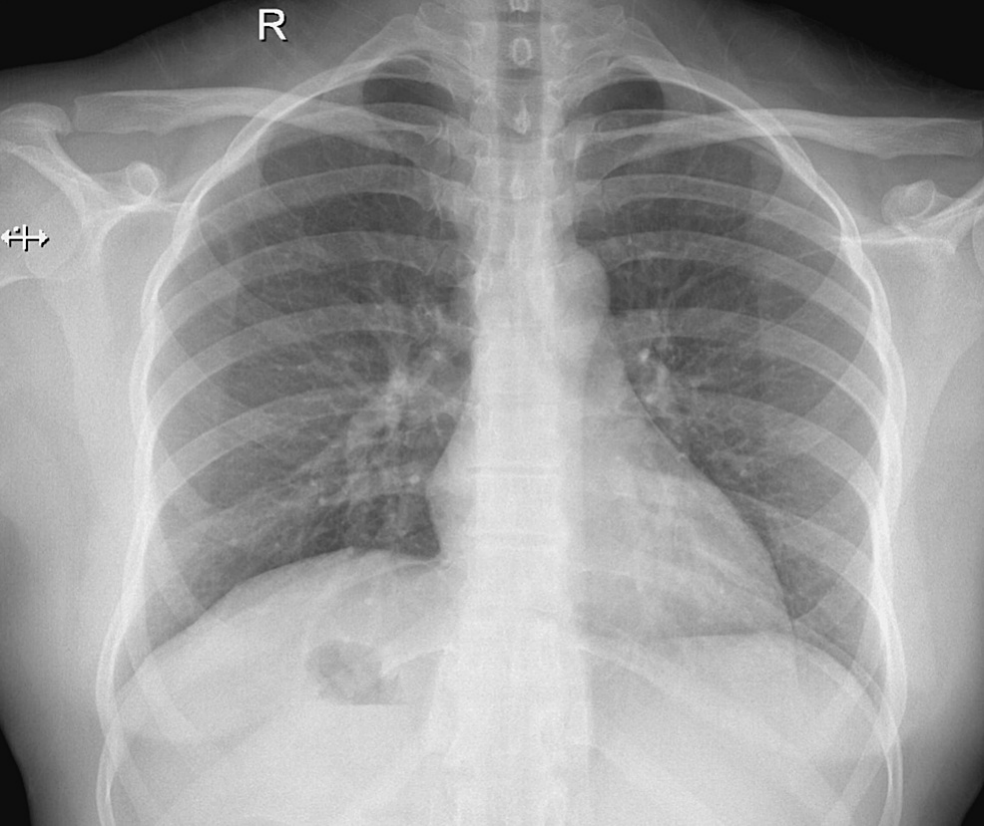
Chest x-ray images of the patient with situs inversus partialis. Gastric gases were noted under the diaphragm on the right side while the heart elements were in their natural positions.

**Figure 2:**
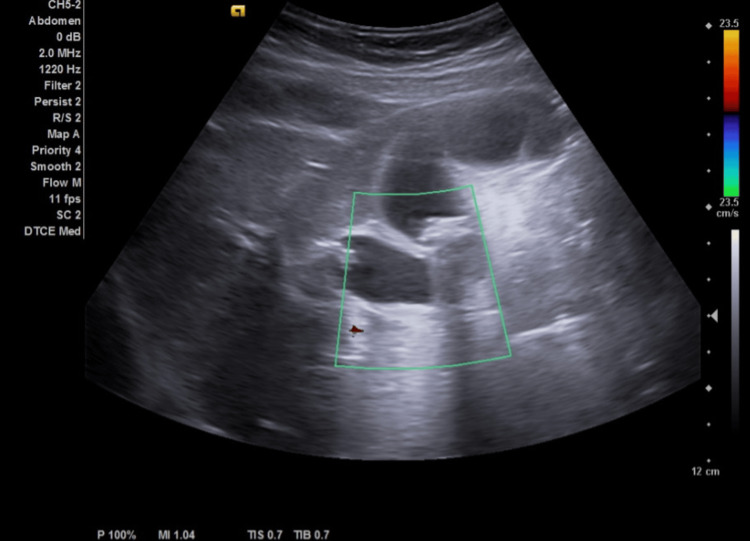
Abdominal ultrasound images of a situs inversus patient with acute cholecystitis. The ultrasound of the 40-year-old female patient indicated gallstones in the gallbladder, with posterior shadowing reflecting the stones.

The examination showed unremarkable cardiac and pulmonary status, along with normal heart sounds and locations. On the abdominal aspect, there was no abdominal distension, scars, or discoloration observed. However, on palpation, there was left hypochondriac tenderness with guarding. On performing the subcostal pressing while breathing the patient stopped and expressed pain (opposite Murphy's sign). The patient has had a chest x-ray and electrocardiogram to exclude any atypical cardiac issues or other emergency events. ECG showed a normal rate and regular rhythm and chest x-ray revealed no cardiac or aortic changes or any increase in lung vascularity due to cardiac malfunction with normal features in all of the thoracic structures and in their natural position. In addition, gastric gases were found on the right side, confirming situs inversus state. The complete blood count (CBC) revealed an 11.34x10^3^/μL white blood cell count (Figure 1). The patient then underwent an ultrasound abdomen on the left upper quadrant, where the gallbladder was identified, showing 15-16 mm gallstones with impaction on the neck of the gallbladder and gallbladder wall thickness of more than 3 mm with minimal pericholecystic edema. The liver showed average-size homogeneous parenchyma, smooth surface, no focal lesion or dilated hepatic biliary radicles (HBRs), and portal vein caliber. The inferior vena cava (IVC) and hepatic veins are not congested. Gallbladder with Phrygian caps folding, multiple large stones (mean: 1.3 cm), diffuse wall thickening, and no gross biliary dilation were noted. The spleen was normal in size and echo pattern. No focal lesions were seen. The pancreas and para-aortic areas were visualized and were grossly remarkable. Both kidneys showed average size, no mass, stone, cyst, or hydronephrosis, and standard parenchymal thickness. The urinary bladder had a normal wall thickness and no stone or group was detected. No further step to computed tomography (CT) scan was made because the diagnosis was established by ultrasound with correlation to the patient’s symptoms and most of the gallbladder stones were isodense, which which would have been missed by CT scan (Figure 2).

The diagnosis of acute cholecystitis in a patient with SIP was confirmed through an abdomen ultrasound, which revealed gallstones with impaction on the gallbladder neck and thickened gallbladder wall, consistent with acute cholecystitis. Additionally, the characteristic clinical signs, including severe epigastric and left hypochondriac abdominal pain exacerbated by fatty food intake, along with left hypochondriac tenderness and an opposite Murphy's sign, further supported the diagnosis. Imaging studies ruled out cardiac abnormalities and confirmed situs inversus, with gastric gases visualized on the right side, consistent with the patient's known condition.

**Figure 3:**
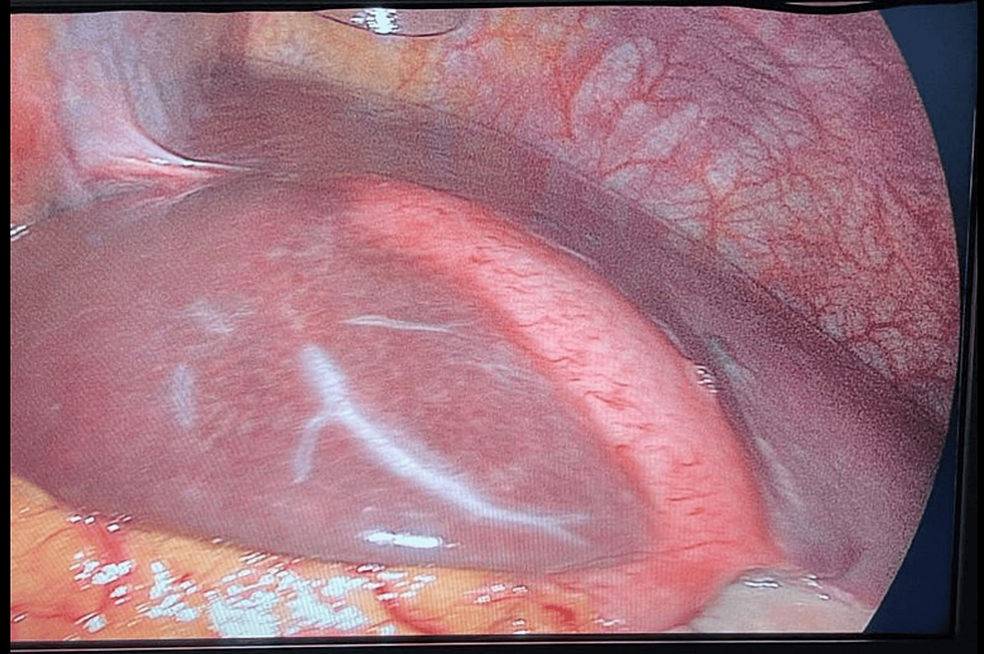
Laparoscopic image of situs inversus patient with acute cholecystitis. The image showed the gall bladder on the anterior aspect of the liver extending from the falciform ligament up to the gallbladder fossa posteriorly.

The patient was hospitalized with acute cholecystitis and started on a treatment plan that comprised fasting and nothing by mouth, as well as the provision of intravenous fluids and drugs. The suggested drugs were IV paracetamol at 1 g three times a day, IV cefuroxime at 750 g three times a day, IV omeprazole at 40 mg once daily, and IV metoclopramide twice a day. The patient subsequently undergoes a laparoscopic cholecystectomy, employing a three-trocar technique with a specular approach. The primary surgeon and camera assistant are positioned on the right side of the patient, while the monitor and first assistant are situated on the left side. This setup facilitates the manipulation of Hartmann's pouch with the left hand through the subxiphoid port and allows for dissection with the right hand through the left midclavicular subcostal ports (Figure 3).

The subsequent examination was done after one day of operation, which showed that the patient's condition was stable and afebrile, and the patient was doing fine with no active complaints. The abdomen was soft and lax, there was mild tenderness over the surgical sites, drain collected around 150 mL of serous fluids. Lab reports showed the WBC was 17, which was acceptable in the post-surgical inflammatory process, with mild elevation of liver enzyme and normal total and direct bilirubin readings. The next plan for the patient was to start a fat-free diet and encourage ambulation. After the second day of the operation, the patient was doing fine, tolerating orally, passing gas and stool, the abdomen was soft and lax, and the drain was 25 mL of serous fluid. All laboratory workup was within normal range. The next plan was to remove the drain and discharge her home after two days of operation with antibiotics and analgesics.

The patient presented to the clinic two weeks later. Surgical wounds were dry and clean, and stitches were removed. Her abdominal examination revealed an improvement in general condition with no active complaints and a soft, lax abdomen. The histopathology report confirmed the expected diagnosis, showing a gross description of an opened gallbladder measuring 7x3x2 cm with ulcerated mucosa. The wall thickness was 0.7 cm, and yellow stones were identified. The microscopic description revealed acute on chronic cholecystitis. The patient was discharged from the clinic.

## Discussion

In this case study, a 40-year-old female with hypothyroidism and situs inversus partialis (SIP) presented with symptoms indicative of acute cholecystitis, including severe epigastric and left hypochondriac abdominal pain, vomiting, and sweating. Clinical examination revealed left hypochondriac tenderness and an opposite Murphy's sign. Imaging studies confirmed situs inversus and identified gallstones with impaction on the gallbladder neck and thickened gallbladder wall. The patient underwent laparoscopic cholecystectomy, and postoperative recovery was uneventful. The histopathology report confirmed acute on chronic cholecystitis. This case underscores the diagnostic challenges posed by altered anatomy in patients with situs inversus partialis and highlights the importance of recognizing characteristic clinical signs and employing appropriate diagnostic modalities for accurate diagnosis and management. The successful outcome of laparoscopic cholecystectomy in this patient demonstrates the feasibility of surgical intervention in such cases.

Acute cholecystitis in patients with SIP presents a complex clinical scenario, necessitating a nuanced approach to diagnosis and surgical management. SIP, characterized by the mirror-image transposition of intraabdominal organs, challenges conventional diagnostic paradigms, as symptoms may manifest atypically [5]. While acute cholecystitis typically presents with right upper quadrant pain, patients with SIP may experience left upper quadrant or epigastric pain due to the aberrant positioning of organs. This divergence from typical symptomatology underscores the importance of a high index of suspicion and comprehensive preoperative evaluation to ensure timely and accurate diagnosis [6].

Leonardo da Vinci, between 1452 and 1519, was the first to document dextrocardia, depicting the heart situated on the right side of the thorax. It wasn't until over a century later that Matthew Baillie formally defined situs inversus, a condition where the chest and abdominal organs are arranged in a perfect mirror image of their usual positions. Situs solitus denotes the typical arrangement of internal organs within the abdomen and chest [7]. Conversely, situs ambiguous or heterotaxy refers to an improper distribution of major internal organs. The estimated incidence of situs inversus is 1:10,000 to 1:20,000 according to Mayo et al. Situs inversus can manifest as situs inversus with dextrocardia (totalis) or situs inversus with levocardia (incompletus), depending on whether the heart is located on the right or left side of the thorax, respectively [8]. It is primarily considered an autosomal recessive genetic disorder, although autosomal dominant and X-linked recessive inheritance patterns have also been observed. While situs inversus totalis and situs inversus incompletus represent the complete reversal and partial reversal of both thoracic and abdominal viscera, respectively, the condition can manifest in various forms due to rare genetic mutations involving more than 20 known genes [7,8].

Apart from left abdominal pain, clinical symptoms of acute cholecystitis in patients with situs inversus (SI) closely resemble those observed in cases with a typical right-sided gallbladder. Diagnosis of SI can be facilitated by identifying irregularities such as an abnormal beat in the right fifth intercostal region or dullness on the left side of the body, which can be confirmed through imaging modalities like ultrasonography, CT, or MRI [9]. However, only about 30% of patients with acute cholecystitis and SI report left upper quadrant or epigastric pain, while 50% of those with a left-side appendix experience left-sided pain. This discrepancy in symptom localization is attributed to the non-transposition of peripheral nervous system components despite the transposition of organs, leading to diffuse abdominal pain. While the incidence of SI equals that of the general population due to insufficient evidence linking it to gallstone development, patients with SI often present with a higher prevalence of gallbladder abnormalities [10].

Preoperative MRI can offer valuable insights during surgical intervention. Although typically asymptomatic, SI may be associated with cardiovascular changes such as atrial situs solitus, discordant ventriculoatrial connection, and discordant atrioventricular connection, alongside gastrointestinal and vascular alterations. Additionally, SI may be linked to syndromes like Yoshikawa syndrome, Kartagener syndrome, Ivemark syndrome, and vena cava obstruction [11]. Kartagener's syndrome, characterized by situs inversus, paranasal sinusitis, and bronchiectasis, affects approximately 25% of individuals with dextrocardia. This condition also includes male sterility and reduced female fecundity, with situs inversus observed in 50% of Kartagener's syndrome cases. Surgical conditions such as appendicitis, cholecystitis, and diverticulitis may affect SI patients similarly to those with normal anatomy, albeit with reversed anatomical positions of symptoms, posing challenges in prompt diagnosis and management [12].

The advent of laparoscopic cholecystectomy has revolutionized the surgical management of gallbladder diseases, offering less invasive approaches and faster recovery times. However, its application in patients with SIP introduces unique technical challenges. The contralateral disposition of visceral organs necessitates adjustments in surgical technique and equipment positioning to navigate the mirror-image anatomy effectively [13]. Surgeons, particularly those who are right-handed, may encounter difficulties in maneuvering instruments and dissecting biliary structures with their non-dominant hand, prolonging surgical time and increasing the risk of iatrogenic injuries. Therefore, meticulous surgical planning and adaptation of techniques are paramount to ensure optimal outcomes in SIP patients undergoing laparoscopic cholecystectomy for acute cholecystitis [14].

Presurgical awareness of SI was crucial in this case, facilitated by in-hospital abdominal ultrasound, heart auscultation, and chest x-ray, enabling adequate surgical planning. Prior to the introduction of laparoscopic techniques in 1991 by Campos and Sipes, conventional open surgery was the primary approach for patients with SI. However, laparoscopic cholecystectomy have since been successfully performed in these patients, with low complication rates attributed to preoperative recognition of the condition. Preoperative evaluation is imperative to rule out any cardiac or gastrointestinal abnormalities that may complicate the procedure [15]. The most widely described technique for laparoscopic cholecystectomy in SI involves utilizing three trocars with a specular approach, positioning surgeons and the camera assistant to the patient's right, and the monitor and first assistant to the left. This setup facilitates optimal manipulation, with the left hand holding Hartmann's pouch through the subxiphoid port and the right hand performing dissection through the left midclavicular subcostal ports. While various laparoscopic procedures have been reported in patients with SI, including cholecystectomy and appendectomy, challenges arise due to the mirror-image anatomy, emphasizing the importance of recognizing anatomical variations and anticipating increased operative times [16].

Despite these challenges, laparoscopic cholecystectomy remains a feasible and effective technique for managing acute cholecystitis in SIP patients. Reports in the literature highlight successful outcomes achieved through innovative approaches and careful surgical execution [17]. Techniques such as utilizing the epigastric port for dissection of Calot’s triangle with the left hand, or positioning the surgeon to the patient's right side, have been proposed to optimize visualization and facilitate precise manipulation of instruments. Furthermore, modifications to port placement, such as caudal displacement of ports, aim to enhance ergonomics and reduce surgeon fatigue, particularly for right-handed surgeons. These strategies underscore the adaptability of laparoscopic techniques to accommodate the anatomical variations presented by SIP, emphasizing the importance of individualized surgical approaches tailored to patient-specific factors [18]. Despite these complexities, our patient underwent laparoscopic cholecystectomy, experiencing postoperative improvement and discharge two days postprocedure, with recommendations for a fat-free diet and ambulation encouragement.

In addition to technical considerations, preoperative assessment plays a crucial role in optimizing surgical outcomes for SIP patients with acute cholecystitis. Comprehensive evaluation, including imaging studies such as ultrasonography, CT scans, and MRI, is essential for confirming the diagnosis of SIP and identifying associated anatomical anomalies that may complicate surgical management [19]. Moreover, a multidisciplinary approach involving collaboration between surgeons, radiologists, and anesthesiologists is essential for comprehensive preoperative planning and intraoperative decision-making [20].

Moving forward, continued research and innovation are needed to further refine surgical techniques and optimize outcomes for SIP patients with acute cholecystitis. Advancements in imaging modalities, such as three-dimensional reconstruction and virtual reality simulation, may enhance preoperative planning and intraoperative navigation, improving surgical precision and reducing operative time. Additionally, the development of specialized instruments designed for use in SIP patients may further facilitate dissection and manipulation of biliary structures, minimizing the technical challenges associated with laparoscopic cholecystectomy in this population [19,20].

## Conclusions

Cholecystitis manifests as inflammation of the gallbladder, presenting a complex scenario during laparoscopic procedures. The gallbladder, an organ prone to various anatomical variations, poses numerous challenges. Additionally, the laparoscopic approach is further complicated by acute manifestations such as edema and heightened vascularity. Situs inversus is a condition that occurs when the chest and abdominal organs are positioned in a perfect mirror image of one another. Except for the left abdominal pain, the clinical symptoms are comparable to those in the right-sided gallbladder. The most common treatment for cholecystitis is laparoscopic cholecystectomy, which is easily accomplished. Reportedly, laparoscopic cholecystectomy has been employed in treating certain cases of situs inversus partialis (SIP). Nevertheless, this procedure remains intricate, notably due to diverse anatomical variations, the presence of acute inflammation, and the dominant hand preferences of surgeons.
